# Mechanisms and Pathological Significance of Liquid–Liquid Phase Separation in Bacteria

**DOI:** 10.1096/fj.202500634RR

**Published:** 2025-09-23

**Authors:** Yanxiao Zhao, Enhui Dai, Mentao Zhang, Yifan Wu, Dongjie Sun, Jiabo Ding

**Affiliations:** ^1^ Institute of Animal Science, Chinese Academy of Agricultural Sciences Beijing China; ^2^ College of Veterinary Medicine, Shandong Agricultural University Taian China

**Keywords:** bacteria, liquid–liquid phase separation (LLPS), mechanisms, pathological significance

## Abstract

Liquid–liquid phase separation (LLPS) has emerged as a fundamental regulatory mechanism in bacterial physiology, orchestrating essential cellular processes including gene expression, stress responses, metabolic homeostasis, and biofilm formation. This phenomenon is driven by intrinsically disordered regions (IDRs), multivalent interactions between modular domains, and dynamic protein‐nucleic acid associations, with precise modulation by environmental parameters such as temperature, ionic strength, and post‐translational modifications (PTMs). The resulting functional condensates confer enhanced environmental adaptability and contribute to antibiotic resistance mechanisms in bacterial populations. These assemblies further impact host‐pathogen interactions through modulation of virulence factor expression and immune evasion strategies, thereby complicating infection management. This comprehensive review systematically examines the molecular mechanisms driving LLPS, its dynamic regulatory networks, and physiological functions in bacteria. We evaluate the therapeutic potential of targeting LLPS pathways for antimicrobial development, with particular emphasis on antibiotic resistance regulation and intestinal commensal colonization. Future research should elucidate the mechanistic roles of LLPS‐associated biomacromolecules in bacterial physiology, characterize their assembly and disassembly dynamics, and explore their therapeutic applications to establish a theoretical foundation for innovative antimicrobial strategies.

## Graphical abstract

LLPS in bacterial infections. In biological systems, the molecular mechanisms underlying LLPS are primarily driven by multivalent protein–protein interactions, protein‐nucleic acid interactions, and intramolecular interactions mediated by IDR. Through LLPS, proteins form condensates that function as ‘microfactories,’ playing crucial roles in regulating bacterial antibiotic resistance, colonization capacity, pathogenicity, stress responses, metabolic regulation, and biofilm formation. Based on these molecular mechanisms, targeting and disrupting LLPS through IDR‐directed approaches or modulation of post‐translational modifications (PTMs) represent a promising therapeutic strategy.

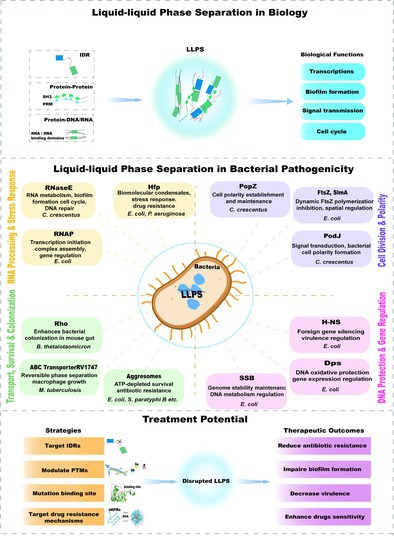

## 1 Introduction

Liquid–liquid phase separation (LLPS) has emerged as a fundamental mechanism governing the assembly and function of membraneless organelles within eukaryotic cells. Unlike prokaryotes, eukaryotic cells exhibit complex structural and functional organization, characterized by a membrane‐bound nucleus containing both membrane‐delimited organelles and diverse membraneless compartments. These membraneless organelles create spatially distinct microenvironments that facilitate selective molecular interactions and enhance biochemical reaction efficiency [1]. The nucleolus was first identified among membraneless organelles [2]. Subsequently, similar membrane‐less organelles were discovered, including stress granules, P bodies, and Cajal bodies [3, 4]. These dynamic assemblies regulate critical cellular processes encompassing gene transcription, signal transduction, and DNA damage repair [5, 6, 7].

In 2009, Hyman et al. first documented the assembly of P granules into droplet‐like condensates through LLPS in *Caenorhabditis elegans* embryonic cytoplasm [8]. This pioneering observation established a new paradigm for understanding membraneless organelle formation and function within subcellular architecture. Subsequent investigations confirmed that diverse membraneless organelles form through LLPS mechanisms [4, 9]. Moreover, several biomolecules, including RNA and proteins, have been shown to promote the formation of these organelles via LLPS. These membrane‐less organelles play essential roles in gene transcription, signal transduction, DNA damage repair, cellular homeostasis regulation, and chromatin structure remodeling [10, 11]. Due to the absence of physical barriers that separate their internal components from the surrounding medium, the concentration gradients and chemical composition stability within these compartments are easily influenced by the external environment. Wang et al. [12] demonstrated that liquid–liquid phase separation is primarily driven by entropy, while enthalpic changes also regulate intermolecular interactions to modulate the phase behavior. This is consistent with the common physical–chemical processes of surfactant assembly and oil–water phase separation. These findings highlight the fundamental challenge of understanding how membraneless compartments concentrate molecules, maintain structural integrity, regulate composition, and modulate internal biochemical activities.

LLPS was initially characterized primarily in eukaryotic systems. However, recent studies have revealed its widespread occurrence in bacteria, thereby expanding our understanding of bacterial intracellular organization. The discovery of LLPS in bacteria originated from observations of intracellular protein aggregates. RNaseE in *Caulobacter crescentus* was the first bacterial protein identified to form LLPS condensates, structurally analogous to eukaryotic P‐bodies (mRNA processing bodies) and stress granules [13], which were considered early evidence of LLPS in bacteria. Subsequent work has continually uncovered further evidence. For instance, Anne‐Marie et al. demonstrated that clusters of bacterial RNA polymerase constitute biomolecular condensates polymerized via LLPS [14], providing crucial insights for investigating bacterial subcellular organization and vital processes. These studies have stimulated extensive research into bacterial LLPS, prompting comprehensive analyses of its biological functions and underlying molecular mechanisms. Such advances have enhanced our understanding of phase separation in prokaryotic physiology while establishing a theoretical framework for developing innovative antimicrobial strategies and therapeutic interventions.

## 2 Molecular Mechanisms and Stability Regulation of LLPS Molecular Mechanisms Driving LLPS

### 2.1 Molecular Mechanisms Driving LLPS

Liquid–liquid phase separation (LLPS) formation is predominantly mediated by multivalent, weak, and reversible interactions between biomacromolecules, particularly proteins and nucleic acids [15, 16]. The principal driving forces can be categorized into three major aspects: IDRs, modular structural domains, and multivalent interactions.

#### 2.1.1 Intrinsically Disordered Domains (IDRs) Drive LLPS

**FIGURE 1:**
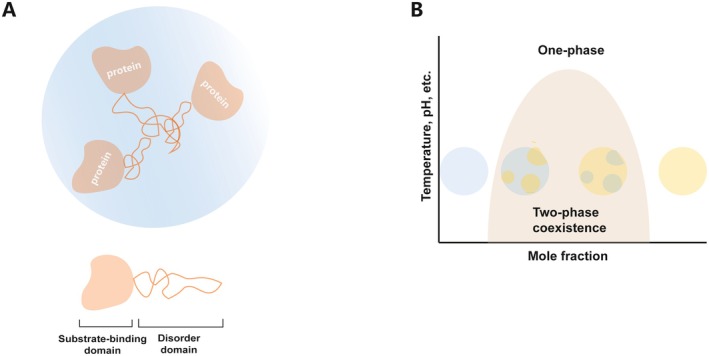
Drivers of phase separation. (A) Phase Diagram of Phase Separation: Horizontal axis (Mole fraction): Represents the mole fraction of molecules, which can be understood as the concentration ratio of different components in the system. Vertical axis (Temperature, pH, etc.): Covers environmental conditions such as temperature and pH, which affect the phase separation process. One—phase: The larger light‐colored area at the top of the figure. When the system is in this area, the molecules are uniformly mixed, presenting a single phase state, with no obvious phase separation phenomenon. Two—phase coexistence: Within this area, the system undergoes liquid–liquid phase separation (LLPS), forming two phases rich in different molecular components. (B) Protein Structure and Function in LLPS: The “disorder domain” (orange stripes) is typically rich in intrinsically disordered regions (IDRs). These disorder domains play a key role in phase separation by driving phase separation through weak interactions with other molecules, promoting the formation and maintenance of aggregates, and serving as an important molecular basis for phase separation of biomolecules.

In protein research, the traditional view holds that amino acid sequence dictates protein structure, which in turn determines function—typically referring to well‐defined three‐dimensional conformations [17]. However, advancing research has identified a class of protein segments termed intrinsically disordered regions (IDRs) that fail to fold into stable structures under physiological conditions [18]. Despite lacking stable tertiary structure and exhibiting high conformational flexibility, IDRs perform essential biological functions [19] and are considered as critical drivers of protein LLPS [20]. This phenomenon occurs because IDRs are enriched in disorder‐promoting amino acids (including Arg, Pro, Glu, Ser, Lys) and contain low‐complexity sequences, facilitating weak intermolecular interactions that drive LLPS [21]. Recent studies have demonstrated that proteins containing IDRs can undergo LLPS under physiological conditions, thereby enhancing interactions and stability within protein complexes [22] (as shown in Figure 1A).

A study concerning *Drosophila C* virus revealed that the IDR1 domain of the immune signaling protein Cactin is critical for phase separation; deletion of the IDR1 region abolished Cactin's LLPS behavior [5]. Furthermore, IDR‐mediated LLPS exhibits amino acid sequence dependency, as site‐specific mutations of aromatic or charged residues disrupt phase separation in FET family proteins [7]. However, IDRs are not essential for protein LLPS. Investigations into the innate immune evasion mechanisms mediated by the SARS‐CoV‐2 nucleocapsid protein (SARS2‐NP) revealed that while the protein contains three distinct IDRs, the dimerization domain is the critical determinant for LLPS. Knocking out the three IDRs separately does not affect the occurrence of LLPS [23].

Given the central role of IDRs in mediating LLPS, accurate identification and prediction of IDRs within proteins have become an essential prerequisite for elucidating their functional mechanisms. Multiple bioinformatics tools, like DisProt (a dedicated database for disordered proteins/domains providing sequence, structural, and functional annotations) and PONDR (predictive algorithms for efficient prediction of disordered regions), facilitate IDR prediction and annotation. Details of these tools are summarized in Table 1, providing researchers with convenient resources for systematic analysis of IDR characteristics and functions.

**TABLE 1:** IDR Prediction Websites.

Website name	Web address	Brief
DisProt	https://www.disprot.org/	DisProt is a specialized database for collecting and annotating intrinsically disordered proteins (IDPs) and disordered structural domains (IDRs). It provides a wealth of annotated information on the sequence, structure, and function of the proteins as well as related literature
DisEMBL	https://sourceforge.net/projects/disembl/	DisEMBL is a Hidden Markov Model (HMM)‐based prediction tool specialized in predicting disordered regions in proteins
PONDR	https://www.pondr.com/	PONDR (Predictor of Naturally Disordered Regions) is a widely used predictor of disordered structural domains that provides a variety of prediction algorithms
MobiDB	https://mobidb.org/	MobiDB is a comprehensive unorganized structured domain database and prediction tool that provides rich annotation information and multiple prediction methods
PrDOS	https://prdos.hgc.jp/cgi‐bin/top.cgi	PrDOS is a support vector machine (SVM)‐based disordered structural domain prediction tool specialized for predicting disordered regions in proteins
D2P2	https://d2p2.pro/about	D2P2 is a comprehensive disordered protein prediction database that integrates six prediction tools, including VL‐XT, VSL2b, and PrDOS, as well as SCOP domain annotations predicted by the SUPERFAMILY predictor for all (mostly structured) SCOP domains, covering 1765 complete proteomes

#### 2.1.2 Interactions Mediated by Proteins With Modular Domains

Proteins containing modular domains are characterized by structures divisible into multiple independent functional modules or domains. A quintessential example involves the interaction between SH3 domains and proline‐rich motifs (PRMs), which can undergo phase separation to form liquid droplets. The strength and specificity of such interactions can be modulated by increasing the number, valency, and binding affinity of receptors and ligands [24]. This phenomenon is also observed in neuronal postsynaptic densities (PSDs), where introducing binding repeat sequences of distinct target protein domains under low protein concentrations (without pre‐existing condensates) promotes the formation of protein‐enriched droplets and reduces the concentration threshold required for LLPS [25].

#### 2.1.3 Multivalent Interactions Drive LLPS

While many fundamental questions regarding the organizational principles and physicochemical driving forces of LLPS remain to be clarified, in many cases, weak, reversible multivalent interactions between proteins and nucleic acids have been established as critical drivers of biomolecular condensates [15].

Li et al. proposed that multivalent interactions are a critical factor in biomolecular phase separation [24]. This viewpoint suggests that biomolecular condensates are composed of numerous multivalent molecules, which form stable aggregates through various elements that control intermolecular or intramolecular interactions. For example, the bacterial polar protein PopZ creates diffusion barriers via selective condensation, such as the signaling protein CtrA, thereby restricting the mobility of cytoplasmic proteins at cell poles [26]. Furthermore, these interactions are typically facilitated by proteins with multivalent modular domains and IDRs [27].

The cytosol represents a highly crowded environment where macromolecules (e.g., proteins, nucleic acids, polysaccharides) must jostle and compete to execute biological functions [28, 29]. In vitro studies demonstrate that adding inert crowding agents frequently induces or enhances LLPS. These agents simulate systems with high viscosity and low diffusion coefficients characteristic of biochemical reactions, thereby enabling exploration of factors like pH, temperature, and ionic strength [28, 30, 31].

Under specific in vitro conditions, LLPS represents an energetically favorable process that is governed by the concentration of macromolecules in aqueous solutions. It is further influenced by macromolecular properties, including polymer length, hydrophobicity, and charge distribution, as well as ambient biophysical conditions such as temperature, pH, and ionic strength [23, 32]. Consequently, LLPS aggregates can form reversibly in response to cellular environmental cues and coexist within the intracellular milieu (Figure 1B).

### 2.2 Regulatory Mechanisms for the Stability of LLPS

The occurrence and state transitions of LLPS are tightly regulated by multiple factors, which include the concentration, physicochemical properties of molecules in solution, and environmental conditions (temperature, pH, salt ion concentration). In addition, post‐translational modifications (PTMs) and molecular chaperones have been identified as regulators of LLPS dynamics [33, 34].

#### 2.2.1 Regulation of LLPS by Environmental Factors

ELF3 has been identified as a temperature‐responsive protein in *Arabidopsis thaliana* that senses environmental temperature changes through its prion‐like domain (PrD), which mediates LLPS. At low temperatures, ELF3 exhibits diffuse intracellular distribution but forms puncta at elevated temperatures. These puncta reversibly dissolve when temperatures decrease [35]. Several proteins, including FUS, TDP‐43, BrD4, Sox2, and Annexin A11, have been reported to undergo LLPS at low salt concentrations but not at intermediate ionic strengths. Intriguingly, exposure to high salt conditions restores LLPS in these proteins, suggesting that hydrophobic and non‐ionic interactions drive LLPS under high‐salt conditions [36]. Interestingly, exposure to high salt conditions can restore LLPS in these proteins. Therefore, hydrophobic and non‐ionic interactions are believed to facilitate LLPS [36]. Another factor influencing LLPS is ATP. In the case of FUS protein, elevated ATP concentrations have been shown to reduce the availability of arginine residues for intermolecular interactions. This leads to disruption of cross‐linking within the protein network, ultimately inhibiting LLPS of the protein solution [37].

#### 2.2.2 Regulation of LLPS by Post‐Translational Modifications (PTMs)

Post‐translational modifications (PTMs) of proteins, including acetylation, ubiquitination, and methylation, play pivotal roles in regulating LLPS. For instance, RNF168, an E3 ubiquitin ligase involved in DNA double‐strand break repair, can undergo SUMOylation (small ubiquitin‐like modifier modification) within the nucleus. This modification promotes LLPS and modulates the efficiency of non‐homologous end joining (NHEJ) repair. Moreover, it enhances the chemosensitivity of colon cancer cells [38]. O‐GlcNAcylation of the RNA‐binding protein EWS has been demonstrated to reduce LLPS occurrence while enhancing protein aggregation dynamics [39]. In the context of antiviral innate immunity, site‐specific acetylation of the DNA‐binding domains (DBDs) of interferon regulatory factors IRF3/IRF7 inhibits LLPS [40].

#### 2.2.3 Stabilization and Remodeling by Molecular Chaperones

In recent years, the critical roles of various molecular chaperones in regulating cellular LLPS have gained increasing recognition. Among these chaperones, heat shock proteins (HSPs) exhibit remarkable chaperone activity and play essential roles in maintaining intracellular protein homeostasis. Specifically, Hsp40, Hsp70, and Hsp90 have been detected in stress granules (SGs), which are known to participate in the regulation of cellular processes [41]. Further investigations revealed that Hdj2 and Hdj1, members of type I and type II Hsp40 proteins respectively, can undergo LLPS in vitro, which is critical for cytoplasmic SG formation. Moreover, Hdj1 has been shown to interact with FUS, a pathogenic protein in the neurodegenerative disease amyotrophic lateral sclerosis (ALS), driving and stabilizing LLPS condensates even under highly dynamic conditions [42]. Additionally, under standard in vitro experimental conditions, Hsp27 effectively suppresses the LLPS and liquid‐to‐solid phase transition (LSPT) of FUS in ALS models. However, phosphorylation by MAPKAPK‐2/3 attenuates the inhibitory effect of Hsp27 on FUS protein LLPS and promotes co‐phase separation of Hsp27 and FUS under stress and other conditions [43].

## 3 Biological Functions of LLPS in Bacteria and Regulation of the Host

**FIGURE 2:**
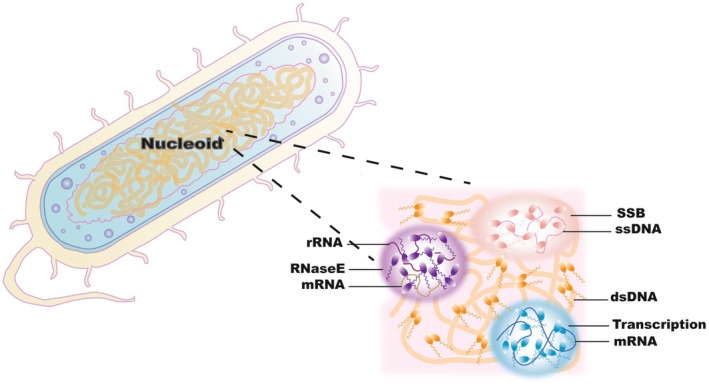
Phase separation in bacteria. The left side depicts the overall structure of a bacterium. The nucleoid, a region where the bacterial genetic material is concentrated, lacks a surrounding nuclear membrane and exists as coiled nucleic acid filaments. On the right side, a close—up view of the nucleoid reveals the liquid—phase separation of various proteins Transcription: Dynamic condensates composed of RNA polymerase (RNAP) and other transcription factors, many of which contain IDRs. Enzymes like RNA polymerase take part in the transcription process using DNA (dsDNA, double—stranded DNA) as a template to synthesize mRNA (messenger RNA). This is the initial step of gene expression, transferring genetic information from DNA to RNA. RNase E: RNase E proteins contain several structural domains that can undergo phase separation, and the interaction between the catalytic domain at the N‐terminal end and the regulatory domain at the C‐terminal end can cause conformational changes when RNase E proteins bind to specific RNA substrates, which in turn can promote the occurrence of phase separation. SSB compartments: Single‐stranded DNA‐binding proteins (SSBs) form tetramers around single‐stranded DNA with prominent intrinsically disordered junctions, which are capped by conserved C‐terminal peptide motifs.

Liquid–liquid phase separation (LLPS) in bacteria has emerged as a prominent research focus, attracting considerable attention from the scientific community. Mounting evidence demonstrates that diverse bacterial proteins leverage LLPS to form membraneless organelles or condensates. Functioning as “microfactories” within bacterial cells, these compartments precisely orchestrate physiological processes essential for survival, proliferation, and environmental adaptation (Figure 2) [44, 45].

Liquid–liquid phase separation (LLPS) in bacteria not only plays crucial roles in bacterial physiological processes but also profoundly impacts host health and disease states. Through LLPS, bacteria regulate processes such as drug resistance, colonization ability, pathogenicity, stress response, metabolic regulation, and biofilm formation, all of which significantly affect the host's health [46]. For instance, proteins such as HslU, Kbl, and AcnB form “aggresomes” via LLPS, enhancing bacterial resistance to antibiotics and increasing the difficulty of treating infections in the host [47]. The Rho protein in *Bacteroides fragilis* regulates gene expression through LLPS, promoting colonization in the host gut and impacting the host's gut microbiota balance and immune function [6]. The H‐NS protein regulates bacterial pathogenicity through LLPS, helping bacteria evade host immune surveillance and leading to more severe infection symptoms [48]. The Dps protein protects DNA from oxidative damage through LLPS, enhancing bacterial stress response in the host, which increases the risk of chronic infection [49, 50, 51]. The ABC transporter Rv1747 regulates bacterial metabolism via LLPS, potentially competing with the host for nutrients and affecting the host's nutrient absorption [52]. RNaseE regulates biofilm formation through LLPS, enhancing bacterial resistance to host immune attacks and antibiotics, leading to chronic or recurrent infections [13]. Therefore, phase separation in bacteria not only increases the risk of host infection and treatment difficulty but may also affect the host's immune function and metabolic health. A deeper understanding of its mechanisms is crucial for developing new antimicrobial strategies and therapeutic approaches.

In summary, LLPS plays multifaceted and critical roles in bacterial physiology and exerts profound effects on host health and disease states. To systematically present these functions, Table 2 provides a detailed list of bacterial proteins with LLPS characteristics, including their identities, bacterial species, biological functions, and associated functional domains.

**TABLE 2:** Bacterial proteins characterized by liquid–liquid phase separation and their functions.

Protein name	Bacteria species	Functions of LLPS	Functional domain of LLPS	References
RNaseE	*Caulobacter crescentus*	RNaseE phase separation plays important roles in bacterial RNA metabolism, gene expression regulation, stress response, cell polarity maintenance, biofilm formation, cell cycle regulation, and DNA repair. It helps bacteria survive and reproduce in complex and changing environments	S1 domain, 5′‐sensor domain, C‐terminal domain, N‐terminal catalytic domain	Al‐Husini et al. [13]
Hfq	*Escherichia coli* *Pseudomonas aeruginosa*	Forms biomolecular condensates by interacting with RNA under stress conditions, playing important roles in bacterial stress response, gene expression regulation, and maintenance of cellular homeostasis Regulate drug resistance‐related gene expression and stress responses	N‐terminal domain, central domain, C‐terminal domain	Goldberger et al. [53], Bloch et al. [54]
ABC Transporter Rv1747	*Mycobacterium tuberculosis*	Its regulatory module exhibits reversible phase separation and is enhanced by phosphorylation by multiple Mtb serine/threonine kinases, which is critical for macrophage growth	ATP‐binding domain (NBD), transmembrane domain (TMD), linker region between NBD and TMD	Heinkel et al. [52]
PodJ	*Caulobacter crescentus*	Mainly involved in the regulation of signal transduction, bacterial cell polarity formation, and coordination of asymmetric cell division	IDR and CC4‐6 domains	Tan et al. [55]
aggresomes	*Escherichia coli* *Salmonella paratyphi B*, *Proteus vuigaris*, *klebsiella pneumoniae*, *Yersinia enterocolitica*, *vibrio parahaemolyticus*, *yersinia pseudotubercuiosis*, *shigella flexneri*, *Mycobacterium smegmatis*	Formation of aggregates promotes bacteria survival under ATP‐depleted conditions play a key role in bacterial response to adversity and antibiotic resistance	ATP‐binding domain	Jin et al. [47]
Rho	*Bacteroides thetaiotaomicron*	Enhances bacterial colonization ability in the mouse gut	IDR	Krypotou et al. [6]
FtsZ、SlmA	*Escherichia coli*	The LLPS of FtsZ and SlmA dynamically inhibits FtsZ polymerization, regulates its spatial distribution, and responds to environmental signals, thereby exerting a multi‐level influence on bacterial division	SBS	Monterroso et al. [31]
PopZ	*Caulobacter crescentus*	Participates in the establishment and maintenance of bacterial cell polarity and regulates the bacterial cell cycle process	Oligomerization domain (OD) at the C‐terminus	Lasker et al. [56]
RNAP	*Escherichia coli*	Accelerates the assembly of transcription initiation complexes, precisely regulates gene expression levels, and stabilizes the intracellular environment	α‐helical domain, RNA‐binding domain, N‐terminal domain	Ladouceur et al. [14]
SSB	*Escherichia coli*	Plays important roles in maintaining genome stability, regulating DNA metabolism, and responding to cellular environmental changes	Intrinsically Disordered Linker (IDL) and C‐terminal Peptide (CTP)	Harami et al. [30]
Dps	*Escherichia coli*	Protects DNA from oxidative damage, regulates gene expression, and participates in bacterial stress responses	N‐terminal and C‐terminal domains	Gupta et al. [50], Gupta and Guptasarma, [49], Sasazawa et al. [51]
H‐NS	*Escherichia coli*	Silences foreign gene expression, maintains genome structure stability, and participates in bacterial virulence regulation	Oligomerization domain at the N‐terminus and DNA‐binding domain at the C‐terminus	Lukose et al. [48]

## 4 LLPS‐Mediated Pathogenic Mechanisms in Bacteria

LLPS refers to the process in which biomacromolecules within cells form membrane‐less organelles or condensates with specific functions. In recent years, the phenomenon of phase separation in bacteria has been closely linked to the occurrence and development of various bacterial diseases, providing new perspectives for the study of bacterial infections.

### 4.1 LLPS‐Mediated Drug Resistance in Bacteria

Recent studies have demonstrated that various pathogenic bacteria form biomolecular condensates to cope with host environmental stresses, thereby enhancing antibiotic resistance, virulence, and infectivity. Dai et al. revealed that biomolecular condensates establish ion gradients between the *Escherichia coli* cytoplasm and condensate interior through selective ion partitioning, resulting in altered membrane potential. These membrane potential changes affect antibiotic transmembrane transport, consequently altering bacterial susceptibility to antibiotics, and providing innovative directions for cellular physiology and disease treatment [57].

The ABC transporter Rv1747 of *Mycobacterium tuberculosis* regulates lipid metabolism through phosphorylation‐mediated LLPS. This protein forms droplet‐like structures within macrophages, dynamically modulating interactions between its nucleotide‐binding domain (NBD) and transmembrane domains (TMDs) to preferentially transport host cholesterol into bacterial cells. Notably, LLPS of Rv1747 correlates strongly with intracellular growth capacity—deletion of Rv1747 results in *M. tuberculosis* growth defects in both macrophages and infected mice [52]. This phase separation mechanism may provide nutritional advantages to *mycobacteria* by concentrating metabolic enzymes to create localized high‐concentration microenvironments and accelerating transmembrane transport of sterols.

A pivotal breakthrough in bacterial LLPS emerged from the Bai laboratory, demonstrating that membrane‐less organelle aggresomes formed via LLPS are critical for environmental stress response and antibiotic resistance [47]. Through analyzing the protein composition of aggresomes, they identified HslU, Kbl, and AcnB as biomarkers of aggresomes. Artificially inducing aggresomes by overexpressing HokB protein to deplete ATP. These aggresomes were observed to exhibit droplet‐like morphology, with internal protein molecules displaying notable fluidity. Single‐molecule fluorescence tracking further revealed heterogeneous dynamics across individual aggresomes. Additionally, mathematical modeling of condensate formation processes enabled the determination of key kinetic parameters, providing quantitative insights into the underlying mechanisms of condensate assembly.

The results verify that strains lacking aggresomes exhibited significantly increased mortality under antibiotic and bacteriophage challenge compared to wild‐type strains, indicating that these aggresomes are indispensable for bacterial antibiotic resistance and other critical biological functions. This discovery provides novel perspectives and potential therapeutic targets for investigating bacterial resistance mechanisms and developing innovative antimicrobial strategies.

Recent studies have demonstrated that Hfq, a highly conserved RNA chaperone protein ubiquitously present in Gram‐negative bacteria, participates in diverse cellular processes including RNA metabolism (mRNA stability and sRNA regulation), DNA compaction, membrane interactions, and amyloid fiber formation. Its structure comprises an N‐terminal RNA‐binding domain, a C‐terminal dimerization domain, and a flexible linker region, enabling biomolecular condensate formation through multivalent interactions [58, 59]. Hfq modulates antibiotic resistance through multiple mechanisms: regulating antibiotic influx via outer membrane proteins (OMPs), controlling flagellar formation, and modulating biofilm matrix production. Hfq modulates antibiotic resistance through multiple mechanisms: regulating antibiotic influx via outer membrane proteins (OMPs), controlling flagellar formation, and modulating biofilm matrix production. Hfq may self‐assemble into dynamic condensates via LLPS, centralizing expression of resistance genes and stress responses. This hypothesis provides a theoretical foundation for developing Hfq‐targeted antimicrobial therapeutics [54].

Collectively, these studies demonstrate that LLPS has emerged as a central strategy employed by bacteria to cope with host immune pressure, antibiotic treatment, and environmental stressors. The development of small‐molecule inhibitors targeting key LLPS regulators represents a promising approach to overcome bacterial adaptive resistance.

### 4.2 Studies on LLPS in Symbiotic Bacteria

Krypotou et al. [6] demonstrated that the IDR of the transcription termination factor Rho in *Bacteroides thetaiotaomicron* plays a critical role in LLPS. Under nutrient‐limited conditions, Rho IDR‐mediated LLPS regulates the expression of hundreds of genes, significantly enhancing bacterial adaptability and colonization capacity within the complex intestinal environment. Wild‐type Rho exhibited substantially higher termination efficiency than IDR‐deleted Rho under LLPS conditions, while both show comparable efficiencies without LLPS. Notably, for certain transcriptional templates, termination occurs exclusively under phase separation conditions, while for others, termination proceeds under both LLPS and non‐LLPS states. These findings establish Rho LLPS as a critical determinant of efficient transcription termination. This discovery provides novel insights into the survival mechanisms and gene regulatory networks of *B. thetaiotaomicron* in the gut, establishing a theoretical foundation for future investigations.

= Rho‐mediated LLPS represents an essential molecular mechanism underlying successful colonization of *B. thetaiotaomicron* and potentially other symbiotic bacteria in the mammalian intestine, with its IDR being indispensable for bacterial gene regulation.

While the discovery of Rho LLPS represents a critical breakthrough in understanding the mechanisms of symbiotic bacterial colonization, significant knowledge gaps remain to be addressed. First, the universality of this mechanism remains unclear. LLPS mediated by Rho‐IDR has been confirmed only in *B. thetaiotaomicron* . However, the gut symbiotic microbiota encompasses thousands of bacterial strains, including Bifidobacterium, Lactobacillus, Clostridium, and numerous others, none of which have been investigated for analogous LLPS mechanisms. Research on Rho LLPS in *B. thetaiotaomicron* has unveiled new perspectives on “biophysical regulatory mechanisms” in symbiont‐host interactions. Nevertheless, bridging the gaps from single‐strain mechanisms to community‐level universality, and from molecular structures to clinical applications, requires addressing numerous research challenges.

### 4.3 Therapeutic Potential of LLPS in Bacterial Infections

Given the complexity of LLPS, diverse molecular strategies have been developed for therapeutic intervention, including small molecules, antibodies, and synthetic peptides. These agents modulate LLPS through multiple mechanisms: targeting IDR, causing binding domains to mutate, or regulating post‐translational modifications.

Yan et al. developed a multi‐targeting antimicrobial strategy that induces global disruption of intracellular LLPS through ligand‐receptor interactions, thereby modulating bacterial subcellular organization and effectively inhibiting the development of antimicrobial resistance. The specific uptake of dAPM‐1 and its intracellular aggregation disrupt subcellular organization, interfering with essential bacterial functions and inducing membrane rupture [60]. This unique mode of action contributes to the broad‐spectrum antimicrobial activity of dAPM‐1, which effectively targets clinically significant multidrug‐resistant bacteria as well as bacteria with diverse lifestyles. These findings establish dAPM‐1 as a compelling paradigm for advancing the development of resistance‐resistant antimicrobial strategies.

Current research targeting LLPS for antimicrobial drug development remains limited. Given that several traditionally undruggable targets have been found to be regulated by LLPS, designing therapeutics against these previously intractable LLPS‐associated targets represents a promising novel strategy for disease treatment. Wang et al. identified a peptide targeting the dimerization domain that disrupts SARS2‐NP LLPS, demonstrating inhibition of SARS‐CoV‐2 replication and restoration of innate immunity both in vitro and in vivo [23]. Through bioinformatic screening, Girdhar et al. identified AIM4 as a potent inhibitor of TDP‐43 aggregation, subsequently demonstrating that AIM4 binds to the C‐terminal domain of TDP‐43 to suppress its LLPS [61]. Furthermore, modulation of post‐translational modifications (PTMs) in relevant molecules has been shown to effectively regulate LLPS. Liu et al. demonstrated that the steroid receptor coactivator‐3 (SRC‐3) inhibitor SI‐2 effectively suppresses methylation modifications of SRC‐3 and blocks its liquid–liquid phase separation (LLPS) process. Through this mechanism, SI‐2 disrupts the interaction between SRC‐3 and nuclear receptor binding SET domain protein 2 (NSD2), thereby enhancing the therapeutic sensitivity of multiple myeloma patients to bortezomib [62].

Additionally, Cheverton et al. proposed that the formation of protein aggregates may induce antibiotic tolerance by disrupting essential pathways, while aggregates must reach a critical threshold to trigger specific drug tolerance phenotypes [63]. Although LLPS‐based drug design and development remain in their infancy, structure‐based drug design targeting phase‐separating proteins and high‐throughput screening represent promising approaches for developing phase separation modulators.

## 5 Conclusions

LLPS, a widespread cellular phenomenon in bacteria, has emerged as a crucial framework for understanding bacterial intracellular organization, physiological regulation, and host interaction mechanisms. This review systematically examines the molecular drivers of bacterial LLPS, including weak intermolecular interactions mediated by IDR, multivalent binding through modular domains, and dynamic protein‐nucleic acid interactions [15, 16]. Further elucidate the dynamic regulation of these processes by environmental factors (temperature, ionic strength), post‐translational modifications (PTMs), and molecular chaperones [36, 64].

Bacterial membraneless condensates formed through LLPS participate in core cellular processes including gene expression regulation [14], stress response mechanisms (oxidative DNA protection by Dps proteins) [50], lipid metabolism homeostasis [52], and biofilm formation [13]. Notably, LLPS of HslU, Kbl, and AcnB profoundly impacts host health and disease progression by enhancing and modulating virulence [47], promoting symbiotic colonization [6], and regulating pathogenicity [48], thereby providing novel insights into bacterial pathogenesis. Current evidence demonstrates that inhibitors targeting key LLPS molecules can effectively disrupt bacterial functions [23, 54]. Future therapeutic development may leverage high‐throughput screening and computational design to identify small molecules or peptides that disrupt IDR‐mediated LLPS [61], with subsequent validation in multidrug‐resistant bacterial infection models [60].

Although current research has elucidated several functions and mechanisms of LLPS in bacteria, numerous critical questions remain to be addressed. Future investigations must deepen our understanding of LLPS molecular mechanisms, particularly elucidating the synergistic roles of IDRs and modular domains across diverse bacterial physiological contexts and establishing quantitative principles governing multivalent interactions [24]. Deciphering how environmental stressors such as nutrient deprivation and oxidative stress, together with post‐translational modifications, precisely regulates the dynamic assembly and disassembly of LLPS condensates remains a fundamental challenge.

In conclusion, research on bacterial LLPS not only provides a new paradigm for understanding prokaryotic cell biology but also creates opportunities for novel antimicrobial strategies. Through interdisciplinary collaboration and technological innovation, comprehensive exploration of LLPS biological significance and therapeutic potential may provide breakthrough theoretical frameworks and technical solutions for addressing antimicrobial resistance and infectious disease treatment challenges.

## Author Contributions

Yanxiao Zhao summarized the molecular mechanism of liquid‐phase separation and drafted the manuscript. Enhui Dai designed the images. Yanxiao Zhao and Enhui Dai contributed equally to this work, and they are co‐first authors. Mengtao Zhang and Yifan Wu collected and organized the literature. Dongjie Sun and Jiabo Ding supervised the project and edited the manuscript. All authors contributed to this article and approved the submitted version.

## Conflicts of Interest

The authors declare no conflicts of interest.

## Data Availability

No new data.
